# hiPSC Extract Attenuates Senescence-Associated Phenotypes in AB-MSCs In Vitro

**DOI:** 10.3390/cells15211965

**Published:** 2026-10-28

**Authors:** Yuanyuan Pan, Lumiao Zhang, Rongyuan Zhu, Fangfang Sun, Guofeng Wu

**Affiliations:** 1 Department of the First Outpatient, Nanjing Stomatological Hospital, Affiliated Hospital of Medical School, Institute of Stomatology, Nanjing University, Nanjing 210000, China; panyyqi@163.com; 2 Department of Prosthodontics, Nanjing Stomatological Hospital, Affiliated Hospital of Medical School, Institute of Stomatology, Nanjing University, Nanjing 210000, China; 13861279592@163.com (L.Z.); 18252101552@163.com (R.Z.); 3 Digital Engineering Center of Stomatology, Nanjing Stomatological Hospital, Affiliated Hospital of Medical School, Institute of Stomatology, Nanjing University, Nanjing 210000, China

**Keywords:** human alveolar bone-derived mesenchymal stromal cells, cellular senescence, induced pluripotent stem cells extract, osteogenic differentiation, proliferation-associated characteristics

## Abstract

Background: Bone marrow mesenchymal stromal cells (BMSCs) are widely used as seed cells in bone tissue engineering. However, sufficient cell quantities for effective engineering typically require prolonged in vitro expansion of BMSCs, which can lead to cellular senescence after excessive subculturing. Objective: This study aimed to investigate the effects of human induced pluripotent stem cell (hiPSC) extract on replicative senescence, clonogenic ability, proliferation-associated characteristics, and osteogenic differentiation capacity of human alveolar bone-derived mesenchymal stromal cells (AB-MSCs). Methods: AB-MSCs were isolated from alveolar bone fragments discarded during dental implant surgery. Cells were divided into three experimental groups: P3 AB-MSCs (Young), P10 AB-MSCs (senescence-associated, YRS), and P10 AB-MSCs treated with hiPSC extract (YRSiE). Senescence markers, clonogenic ability, proliferation-associated characteristics, osteogenic potential, mRNA sequencing and pathway-related expression changes were compared among groups. Small RNA sequencing on hiPSCs and AB-MSCs with qRT-PCR validation were performed to identify candidate miRNAs in hiPSC extract that were differentially expressed in this setting. Results: In the presence of hiPSC extract, senescent AB-MSCs showed altered morphology, a lower proportion of β-galactosidase-positive cells, higher Ki67 expression, and lower expression of the senescence-associated genes and proteins P16 and P21. Treated senescent AB-MSCs also showed higher proliferation-associated metabolic activity, clonogenicity, and osteogenic differentiation than untreated senescent cells. Pluripotency-related genes (NANOG, OCT-4) and osteogenic markers (ALP, RUNX2, OCN) were higher in the treated group. qRT-PCR validation of small RNA sequencing data showed that miR-514a-3p, miR-582-5p and miR-520c-3p were upregulated in senescent AB-MSCs after hiPSC extract treatment. Concurrently, pathway enrichment and pathway-related expression changes were consistent with an association with the NF-κB pathway. Conclusion: In this in vitro study, hiPSC extract attenuates senescence-associated phenotypes in AB-MSCs, and the observed transcriptional and pathway-related expression changes were consistent with NF-κB pathway enrichment, with the identified miRNAs upregulated in treated cells and previously reported to target NF-κB pathway components. However, these mechanisms require further validation.

## 1. Introduction

The jawbone is one of the most anatomically complex bones in the human body and plays a vital role in maintaining physiological functions such as facial support, aesthetic contour, and articulation [1,2]. Jaw defects resulting from trauma, infection, tumor resection, or aging can lead to significant functional impairment and adversely affect mental health, making their reconstruction an active research focus in dentistry [3]. Current clinical approaches for bone regeneration primarily include autologous and allogeneic bone grafts. However, autografts are limited by donor site availability, while allografts carry risks of infection and immune rejection [4,5]. Therefore, achieving effective bone regeneration remains a major clinical challenge.

Driven by these unmet needs, bone tissue engineering has emerged as a promising alternative, with seed cells serving as a foundational element [6]. A key limitation in this field is the scarcity of ideal seed cells that can reliably orchestrate an osteogenic microenvironment [7]. Bone marrow-derived mesenchymal stromal cells (BMSCs) have attracted widespread attention due to their strong osteogenic potential and have been applied in the treatment of osteoporosis, hematological diseases, and musculoskeletal disorders, as well as in bone tissue engineering [8,9]. However, BMSCs constitute only about 0.01% of bone marrow cells, and both their numbers and differentiation capacity decline with age [3]. Large-scale expansion in vitro is necessary for tissue engineering applications, but long-term culture leads to replicative senescence, characterized by flattened morphology, reduced proliferation, decreased clonogenicity, and diminished osteogenic potential [10,11]. These limitations significantly hinder the clinical utility of BMSCs, making the reversal of replicative senescence a major research priority in bone regeneration.

Several strategies have been explored to counteract MSC senescence, including treatment with drugs, exosomes, growth factors, antioxidants, or extracellular matrix components [12]. iPSCs—derivable from patient somatic cells—offer distinct advantages: ease of access, no immune rejection, minimal ethical concerns, and broad differentiation potential. Recently, exosomes derived from induced pluripotent stem cells (iPSCs) have gained interest as a cell-free therapeutic approach [13]. Compared with conventional cell therapies, iPSC-exosomes offer multiple advantages [14]. Myeongsik Oh et al. reported that iPSC-derived exosomes enhanced proliferation and migration in senescent skin fibroblasts, reduced senescence-associated β-galactosidase activity and MMP-1/3 expression, and restored collagen I synthesis [15]. However, The clinical translation of iPSC-Exos is hampered by several obstacles, including low production yield, limited stability, short half-life, and inefficient delivery [16]. In particular, low yield, instability at room temperature, and challenges in purification, storage, and contamination control constitute major barriers to their widespread use [17]. Exosomes contain a variety of proteins and RNAs, and they generally have a short half-life. Storage temperature is also critical to exosome stability and must be maintained between −20 °C and −80 °C to preserve the expression of exosomal markers [18]. Studies have shown that exosome uptake is associated with intracellular and microenvironmental acidity, indicating that the microenvironment influences the delivery efficiency of exosomes [19]. For factors that act at the intracellular level, delivering them into the correct cellular compartments while maintaining their stability, integrity, and biological potency remains challenging and costly.

In contrast, cell extracts from stem or pluripotent cells offer a more practical alternative. Such extracts have been shown to induce reprogramming of differentiated cells into a pluripotent state [20]. The extraction process is efficient, scalable, and avoids risks associated with exogenous genetic material, showing great promise for clinical application [21]. The unlimited self-renewal capacity of hiPSCs enables the generation of sufficient cell from a single large-scale culture to produce extract for multiple treatments, making the “single-batch” production a non-issue for practical scale-up. Therefore, hiPSC extracts exhibiting significant potential for applications in regenerative medicine and tissue repair. Notably, no prior studies have investigated the effects of hiPSC extract. Recent studies on other cell extracts have shown promising results. Na et al. reported that adipose-derived stem cell extracts accelerated wound healing and enhanced fibroblast proliferation and migration [22]. Oocyte extracts co-cultured with BMSCs promoted an embryonic-like phenotype, increased proliferation, and upregulated pluripotency genes (OCT-4, NANOG, SOX2) while downregulating mesenchymal genes [23]. Labial gland stem cell extracts restored salivary flow rates by 50–60% and functionality in irradiated murine salivary glands [24]. Given that other cell-derived extracts have been reported to restore cellular function and iPSC-derived exosomes have also been shown to exert anti-senescence effects, and considering the inherent limitations of exosomes, we hypothesized that unfractionated hiPSC extract may likewise exert rejuvenating effects.

However, although iPSC-derived exosomes have been shown to exert anti-senescence effects, it remains unknown whether unfractionated hiPSC extract can attenuate replicative senescence in AB-MSCs, and the underlying mechanisms have not been explored. In this study, we leverage the benefits of human iPSCs (hiPSCs) and cell extract technology to investigate whether hiPSC-derived extracts can attenuate senescent AB-MSCs by enhancing their proliferative capacity, suppressing senescence-associated secretory phenotypes, and restoring osteogenic potential.

## 2. Materials and Methods

### 2.1. Isolation and Culture of AB-MSCs

Primary AB-MSCs were obtained from clinically discarded alveolar bone tissues of consenting donors, with approval from the Ethics Committee of Nanjing Stomatological Hospital (Approval No.: NJSH-2022NL-49). Donors included 3 females and 4 males, aged 20–60 years. Briefly, alveolar bone tissues were minced into fragments of less than 1 mm^3^ and seeded into 10-cm dishes (Corning Inc., Corning, NY, USA) with α-MEM (Gibco, Grand Island, NY, USA) containing 15% FBS (Gibco), 0.1 mM L-ascorbic acid 2-phosphate (Sigma-Aldrich, St. Louis, MO, USA), 100 U/mL penicillin, and 100 µg/mL streptomycin (Gibco). Cultures were kept at 37 °C in 5% CO_2_ with medium renewal every 3 days. Upon reaching 80% confluence, cells were detached with 0.25% trypsin/EDTA (Gibco) and passaged. Passage 3 cells showing robust growth were employed in the subsequent experiments. A senescence-associated phenotype was induced by continuous culture to passage 10. All experiments were performed using primary cells from three independent donors (*n* = 3).

This culture is in fact a heterogeneous population of AB-MSCs contains a fraction of bona fide stem cells, together with progenitors and cells at more advanced stages of differentiation. At present, no specific marker permits the isolation of a pure population of mesenchymal stem cells. Our use of the term “MSCs” follows the current consensus in the field [25,26].

### 2.2. Preparation of hiPSC Extract

hiPSCs (derived from human gingival fibroblasts in our previously published work [27]) were recovered and cultured on Matrigel^®^ Matrix (Hyclone, Logan, UT, USA)-coated 10 cm dishes in NcTarget™ hPSC Medium (Nuwacell, Hefei, China, catalog No. RP01020), a serum-free, feeder-free, clinical-grade formulation specifically designed to support long-term maintenance of hiPSC self-renewal and trilineage differentiation potential. Incubation was carried out at 37 °C in a humidified environment with 5% CO_2_ under normoxic conditions (21% O_2_). When cells reached approximately 90% confluence, they were detached and centrifuged twice, then resuspended in PBS to an appropriate volume. The cell suspension was stored at −80 °C for 24 h, rapidly thawed at room temperature, and gently shaken. This freeze–thaw cycle was conducted a minimum of three times to ensure complete lysis. The supernatant (hiPSC extract) was collected by centrifugation at 12,000× *g* for 15 min at 4 °C. A BCA assay kit (Beyotime, Shanghai, China) was employed to determine the protein concentration. The experimental groups were designed as follows: P3 AB-MSCs (Young), P10 AB-MSCs (YRS), and P10 AB-MSCs treated with hiPSC extract (YRSiE). The YRSiE group was cultured in complete medium supplemented with hiPSC extract for 6–7 days, with medium refreshed every two days. Approximately 3 independent extract batches were used, and treatment was continued during osteogenic induction. To quantitatively support the morphological observations, ImageJ (version 1.54) software was used to measure cell area (μm^2^) in at least 50 randomly selected cells per group (Young, YRS, and YRSiE).

### 2.3. CCK-8 Assay

P10 AB-MSCs were inoculated into 96-well plates at 3 × 10^3^ cells per well, followed by incubation at 37 °C in 5% CO_2_. Cells were treated with 0, 50, 75, 100, 125, 150 µg/mL hiPSC extract after adherence. Fresh medium supplemented with 10% (*v*/*v*) CCK-8 reagent (Beyotime, Shanghai, China) was substituted for the culture medium on days 3, 5, and 7. After incubation at 37 °C for 4 h, absorbance was read at 450 nm using a microplate reader. The optimal concentration for promoting cell proliferation was determined by comparing OD values among groups at different time points.

Cell proliferation-associated metabolic activity was measured by CCK-8 assay, for which cells from the three groups were seeded into 96-well plates at 3 × 10^3^ cells per well. On days 1, 3, 5, and 7, fresh medium containing CCK-8 reagent was added in place of the original medium, and the cells were incubated at 37 °C for 4 h. Each sample was tested in quadruplicate at every time point. Absorbance was measured to assess proliferative capacity.

### 2.4. Colony-Forming Unit Assay

Single-cell suspensions containing 3 × 10^3^ cells were seeded into 10-cm culture plates in α-MEM containing 15% FBS and incubated at 37 °C with 5% CO_2_. After 14 days, the cells were fixed using 4% formalin and subjected to 1% crystal violet staining (Solarbio, Beijing, China). Colonies containing at least 50 cells were counted via ImageJ, with 3 biological replicates per group. Colony formation efficiency was calculated as: (Number of colonies counted/Number of cells seeded) × 100%.

### 2.5. Oil Red O (ORO) Staining

P3 AB-MSCs were cultured in 6-well plates at 100% confluence using adipogenic induction medium. At three weeks after adipogenic induction, hBMSC-derived adipocytes were fixed and subjected to Oil Red O (ORO) staining (OriCell, Guangzhou, China) to visualize intracellular lipid droplets. Stained cells were photographed under a microscope.

### 2.6. Flow Cytometry Analysis

To identify the MSC phenotype, approximately 1 × 10^7^ AB-MSCs were incubated with monoclonal antibodies against human FITC-CD34 (560942, BD Biosciences), FITC-CD45 (555482, BD Biosciences), PE-CD73 (561014, BD Biosciences), and PE-CD90 (555595, BD Biosciences) for 30 min at 4 °C following the manufacturer’s instructions. The unstained tube was used to define the FSC/SSC cell population gate and the background baseline, and the same gating was applied to all sample tubes. The signal distribution of the unstained tube in each fluorescence channel established the autofluorescence background, against which marker positivity or negativity in sample tubes was determined. 20,000 events were acquired per sample. Cell analysis was performed with a BD FACSAria flow cytometer (BD Biosciences, Franklin Lakes, NJ, USA), and data were processed with FlowJo (version 10.9) software.

Ki67 expression was assessed by intracellular flow cytometry. Cells were resuspended in cell staining buffer at a density of 0.5 × 10^6^ cells/mL. A 100-μL aliquot of the cell suspension was incubated with CoraLite^®^ Plus 488-conjugated Ki-67 polyclonal antibody (CL488-27309, Proteintech, Wuhan, China) at a dilution of 1:100 (*v*/*v*) for 30 min at room temperature in the dark. Ki67 expression was reported as mean fluorescence intensity (MFI).

### 2.7. Senescence-Associated β-Galactosidase Staining

SA-β-gal activity was evaluated in the three hBMSC groups using a senescence-associated β-galactosidase staining kit (Beyotime, Shanghai, China). Cells were seeded in 6-well plates at 1.5 × 10^5^ cells per well. Upon reaching ~90% confluence, the medium was removed, and cells were rinsed once with PBS, fixed with 4% paraformaldehyde for 15 min, washed three times with PBS, and then incubated with 1 mL of SA-β-gal working solution per well at 37 °C overnight in the dark. Senescent cells were visualized under an optical microscope, and three random fields per well were imaged. Quantification was performed using ImageJ software.

### 2.8. Alkaline Phosphatase (ALP) Activity Determination, ALP Staining

Cells were cultured in osteogenic induction medium containing α-MEM with 15% FBS, 0.1 mM L-ascorbic acid 2-phosphate (Sigma-Aldrich), 100 U/mL penicillin, 100 μg/mL streptomycin (Gibco), 1.8 mM KH_2_PO_4_ (Sigma-Aldrich), and 10^−8^ M dexamethasone sodium phosphate (Sigma-Aldrich). The medium was replaced every 2–3 days. After 7 days of induction, AB-MSCs were washed twice in PBS and fixed with 4% paraformaldehyde for 15 min, after which they were stained by an alkaline phosphatase (ALP) staining kit (Beyotime, Shanghai, China). ALP activity was quantified with an Alkaline Phosphatase Assay Kit (Beyotime) according to the manufacturer’s protocol.

### 2.9. Alizarin Red Staining

AB-MSCs were cultured in 6-well plates at 100% confluence using osteogenic induction medium. The medium was replaced every three days, and cells were maintained under induction for 21 days. Mineralization was evaluated by Alizarin Red staining (Sigma-Aldrich). For quantification, mineralized nodules were dissolved in 10% cetylpyridinium chloride (Sigma-Aldrich) at room temperature for 20 min. The absorbance of the supernatant was read at 562 nm on a microplate reader in triplicate.

### 2.10. Quantitative Real-Time Polymerase Chain Reaction (qRT-PCR)

Total RNA was extracted from the three groups of AB-MSCs—cultured for 7 days under osteogenic induction or normal conditions—using TRIzol Reagent (Invitrogen, Carlsbad, CA, USA). cDNA synthesis was performed with the Hiscript RT SuperMix kit (Vazyme, Nanjing, China) in accordance with the manufacturer’s protocol. Quantitative real-time PCR was conducted using SYBR qPCR Master Mix (Vazyme, Nanjing, China) on an ABI QuantStudio™ 6 Flex system (Thermo Fisher Scientific, Waltham, MA, USA). Each reaction was run in triplicate. Relative gene expression was determined by the 2^−ΔΔCt^ method, using GAPDH (Sangon Biotech, Shanghai, China) as the internal control. Primer sequences are listed in Table 1.

### 2.11. Western Blot Assay

After 7 days of osteogenic induction or normal conditions, AB-MSCs were lysed on ice using RIPA lysis buffer (Beyotime, Shanghai, China) containing a protease inhibitor cocktail (Thermo Fisher, Rockford, IL, USA). The protein concentration was quantified with a BCA protein assay kit (Beyotime). Proteins were denatured by adding 5× SDS loading buffer (Beyotime) and heated at 95 °C for 5 min. Total protein (30 μg per lane) underwent separation by 10% SDS-PAGE and was then electrotransferred to a PVDF membrane. Following 1 h of blocking in NcmBlot blocking buffer (NCM Biotech, Suzhou, China), the membrane was probed overnight at 4 °C with the following primary antibodies: anti-GAPDH (1:6000; Proteintech, China), anti-RUNX2 (1:1000; CST, Danvers, MA, USA), anti-OCN (1:2000; Proteintech, China), anti-Phospho-NF-κB p65 (82335-1-RR; Proteintech, China), anti-IκBα (10268-1-AP; Proteintech, China), anti-Phospho-IκBα (82349-1-RR; Proteintech, China), anti-P21 (10355-1-AP; Proteintech, China), anti-P16-INK4A (30519-1-AP; Proteintech, China), and anti-β-actin (20536-1-AP; Proteintech, China). After washing, the membrane was incubated with HRP-conjugated secondary antibodies (A0208, A0216; Beyotime, China) for 1 h. GAPDH and β-actin were used as the internal loading control.

### 2.12. mRNA-Seq

Total RNA was extracted from the three groups of AB-MSCs using TRIzol Reagent (Invitrogen, Carlsbad, CA, USA) and used for RNA sequencing. Sequencing data were analyzed on an Illumina platform. Differential expression analysis was performed using DESeq2. Gene expression was normalized using the median-of-ratios method, a linear model was used to estimate gene expression. The Wald test was used to calculate *p*-values for differential expression between the two groups. *p*-values were corrected using the Benjamini–Hochberg method to obtain padj (FDR). Differentially expressed genes (DEGs) were identified based on |log_2_ fold change| ≥ 1 and *q* < 0.05. KEGG and GO pathway analyses were carried out to investigate the functional implications of these DEGs.

### 2.13. Small RNA Sequencing

Small RNA sequencing was performed to profile miRNA expression. Total RNA was extracted from AB-MSCs and hiPSCs using TRIzol Reagent. Sequencing data were subjected to single-end sequencing with a read length of 50 bp (SE50) on an Illumina platform. Known miRNAs were annotated against the miRBase database (Release 22). miRNA expression levels were normalized to reads per million (RPM). RPM-normalized values are directly comparable across different samples, enabling reliable assessment of relative miRNA abundance between groups. Differentially expressed miRNAs were identified with a |log_2_ fold change| ≥ 1 and *q* < 0.05.

### 2.14. Statistical Analysis

Data are presented as mean ± SD from at least three independent experiments. For multi-group comparisons, one-way ANOVA followed by Tukey’s post hoc test was used, and for experiments involving two factors, two-way ANOVA followed by Tukey’s post hoc test was applied. Normality was assessed using the Shapiro–Wilk test, and homogeneity of variance was assessed using Brown–Forsythe test. When these assumptions were violated, non-parametric alternatives (Kruskal–Wallis test for multi-group comparisons) were used. Statistical analyses were performed using GraphPad Prism 9.0 (GraphPad Software, San Diego, CA, USA). The threshold for statistical significance was defined as *p* < 0.05 (* *p* < 0.05, ** *p* < 0.01, *** *p* < 0.001, **** *p* < 0.0001). The statistical unit was the independent biological donor (*n* = 3 donors).

## 3. Results

### 3.1. The Phenotypic Characteristics of AB-MSCs

**Figure 1:**
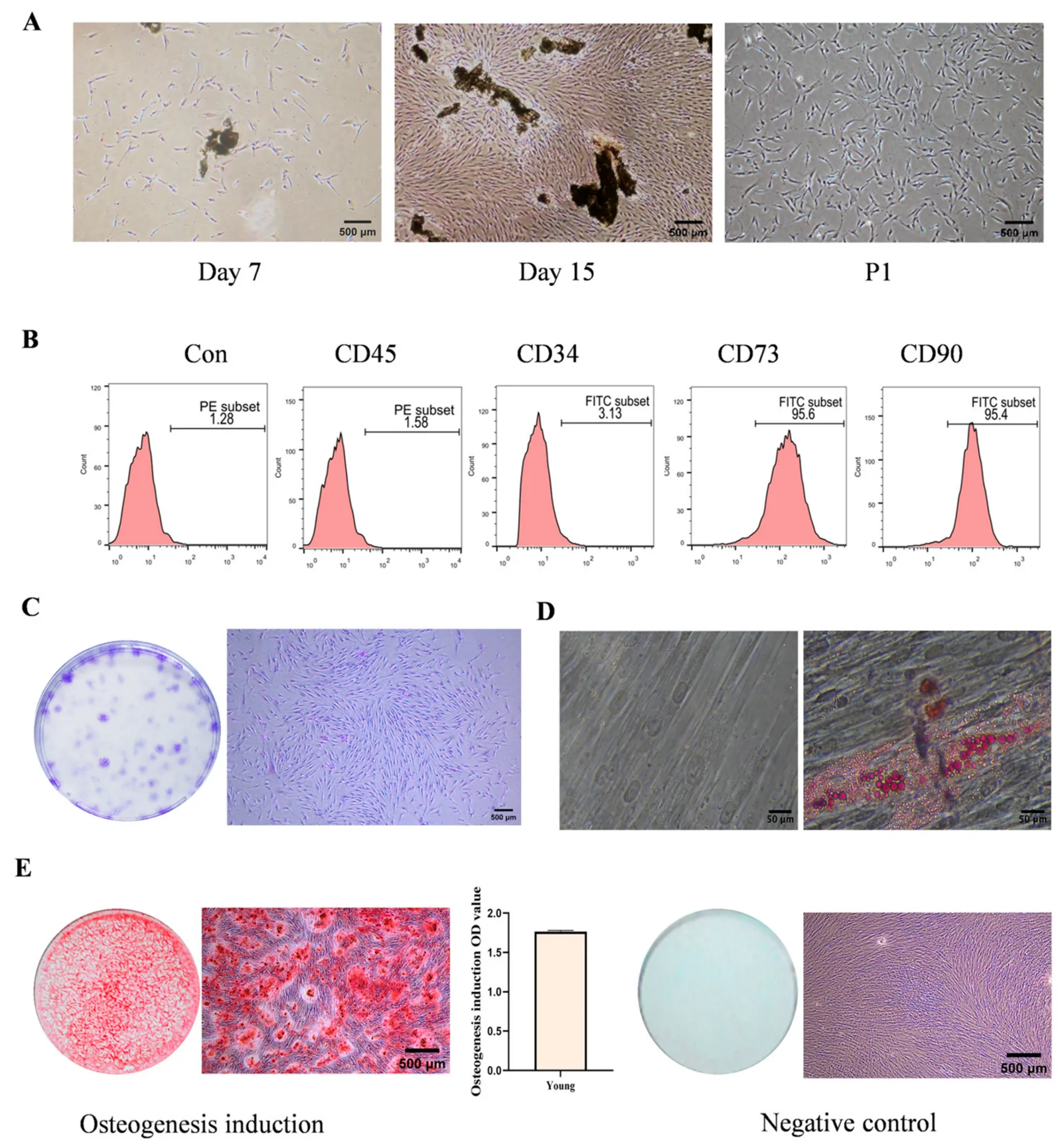
Characterization of isolated AB-MSCs. The following identification of surface markers, clonogenic capacity, osteogenic and adipogenic differentiation potential was performed using P3 BMSCs (Young group). (**A**) Morphology of AB-MSCs at different time points during isolation and culture (scale bar = 500 μm). (**B**) Immunophenotype of P3 AB-MSCs (Young group) assessed by flow cytometry. Cells were positive for CD73 (95.6%) and CD90 (95.4%), and negative for CD34 (3.13%) and CD45 (1.58%). (**C**) Clonogenic ability of P3 AB-MSCs (Young group): general view (**left**) and microscopic view (**right**; scale bar = 500 μm). (**D**) Oil Red O staining showed negligible staining in the negative control (**left**), and abundant lipid droplet accumulation in the adipogenic group (right; scale bar = 50 μm). (**E**) Osteogenic differentiation (**left**) and negative control (**right**) of P3 AB-MSCs (Young group) demonstrated by Alizarin Red staining (scale bar = 500 μm) and semi-quantitative spectrophotometric elution data (OD at 562 nm). *n* = 3 donors. One-way ANOVA with Tukey’s post hoc test.

Primary AB-MSCs were isolated using the explant outgrowth method. By day 7, primary cells were observed migrating from the tissue explants (Figure 1A, Day7). These cells expanded and reached approximately 90% confluence by day 15 (Figure 1A, Day15). After trypsinization and centrifugation, cells were passaged (Figure 1A, P1), displaying a monolayer of adherent, spindle-shaped cells consistent with typical MSC morphology.

Immunophenotypic analysis confirmed that the isolated AB-MSCs expressed characteristic MSC surface markers CD73 and CD90, while lacking expression of hematopoietic markers CD34 and CD45 (Figure 1B).

The AB-MSCs demonstrated clonogenic capacity, forming multiple uniform and well-dispersed colonies that appeared large and dense under microscopy (Figure 1C). Adipogenic differentiation was evaluated by Oil Red O (ORO) staining. After 21 days of induction, cells were fixed and stained with ORO solution. The negative control group (cells cultured in normal growth medium) exhibited negligible lipid droplet formation (Figure 1D, left). In contrast, the adipogenic induction group displayed abundant red-stained lipid droplets within the cytoplasm, indicating successful adipogenic differentiation (Figure 1D, right). Furthermore, mineralization was confirmed by positive Alizarin Red staining after osteogenic induction (Figure 1E, left), semi-quantitative spectrophotometric elution data (OD at 562 nm) indicating successful osteogenic differentiation. Non-differentiated negative control cells cultured in proliferation medium showed minimal to no Alizarin Red staining (Figure 1E, right).

### 3.2. Impact of hiPSC Extract on the Phenotypic Characteristics of Senescent AB-MSCs

**Figure 2:**
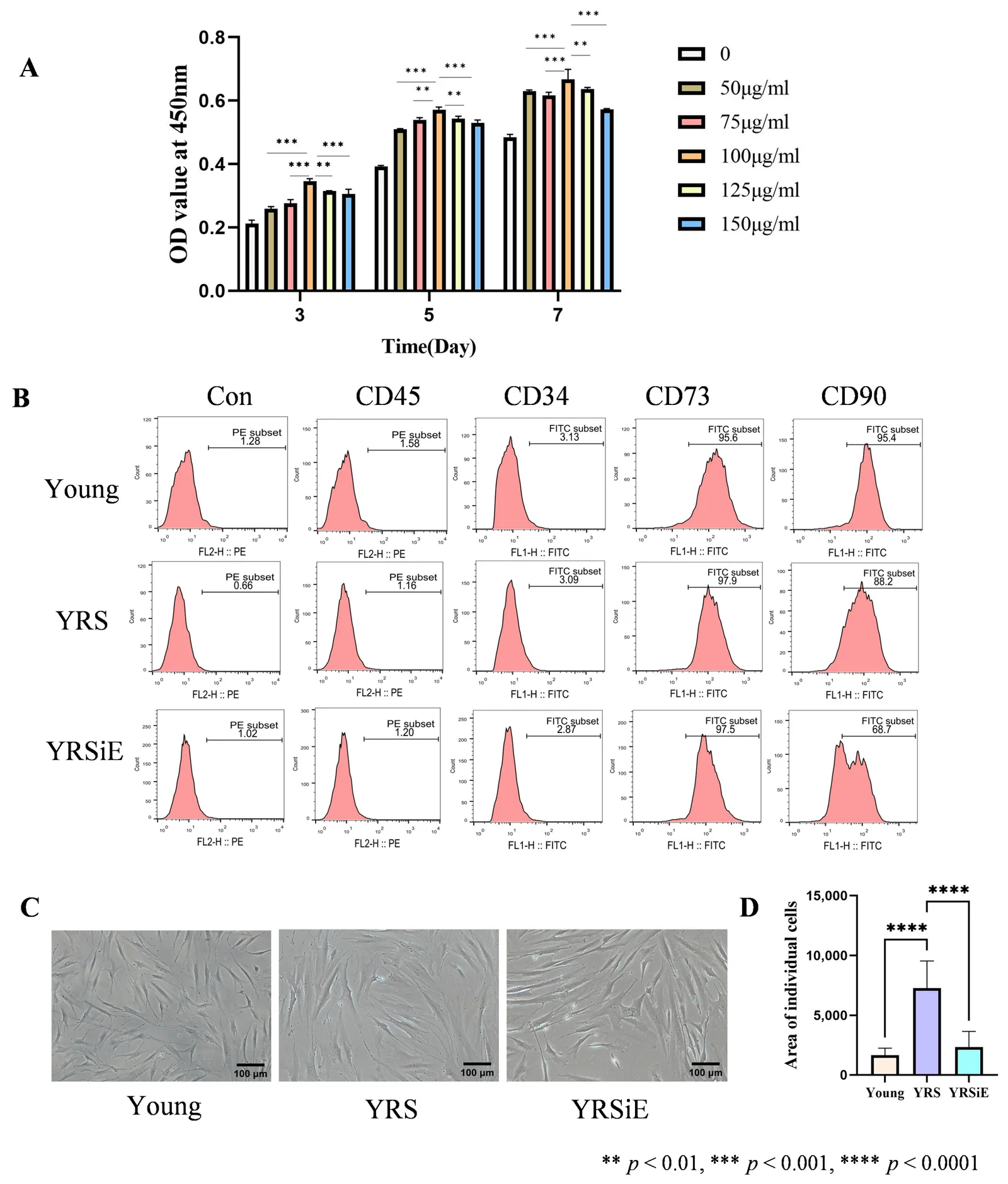
Effects of hiPSC extract on hBMSC immunophenotype and morphology. (**A**) Effects of graded concentrations of hiPSC extract (0–150 µg/mL) on hBMSC proliferation-associated metabolic activity assessed by CCK-8 assay at days 3, 5, and 7. (**B**) Expression of MSC-positive markers (CD73, CD90) and hematopoietic-negative markers (CD34, CD45) in each group, as determined by flow cytometry. The Young panel uses the same P3 characterization data as shown in Figure 1B. (**C**) Morphological changes in AB-MSCs across experimental groups. Scale bar: 100 μm. (**D**) Quantitative analysis of cell morphology. *n* = 3 donors. Two-way ANOVA (**A**) and one-way ANOVA (**B**–**D**) with Tukey’s post hoc test.

CCK-8 assay revealed that hiPSC extract promoted hBMSC proliferation-associated metabolic activity in a concentration-dependent manner within the range of 0–150 µg/mL, with the maximum effect at 100 µg/mL on days 3, 5, and 7 (Figure 2A). Based on these results, 100 µg/mL was selected as the optimal concentration for all subsequent experiments.

All three groups showed expression of MSC surface markers (CD73 and CD90) and absence of hematopoietic markers (CD34 and CD45) in Figure 2B, indicating that hiPSC extract treatment did not alter the fundamental MSC immunophenotype. YRSiE cells showed a shift in CD90 positivity (68.7%) compared with YRS cells (88.2%) and Young cells (95.4%). The decrease in CD90 positivity should not be interpreted as a loss of MSC identity, as CD90 expression is known to vary with donor, passage, culture conditions, and cellular state, and CD90-low MSC subpopulations have been reported to retain MSC characteristics and exhibit enhanced osteogenic potential [28], consistent with the improved osteogenic differentiation observed in YRSiE cells in our subsequent experiments. This shift is interpreted as a treatment-associated change in surface marker distribution rather than a loss of MSC identity, and its significance requires further characterization. Morphologically, P3 AB-MSCs in the Young group exhibited a long, spindle-shaped fibroblast-like appearance. In contrast, P10 cells in the YRS group showed a flattened, enlarged, and irregular morphology. Notably, hiPSC extract treatment in the YRSiE group restored a smaller, more spindle-shaped morphology resembling that of younger cells (Figure 2C). Quantitative analysis of cellular morphology demonstrated that the cell area in the YRSiE group was significantly decreased compared with the YRS group, and was restored to a level comparable to that of the Young group (Figure 2D).

### 3.3. hiPSC Extract Enhances the Growth of AB-MSCs and Attenuates the Senescence of AB-MSCs

**Figure 3:**
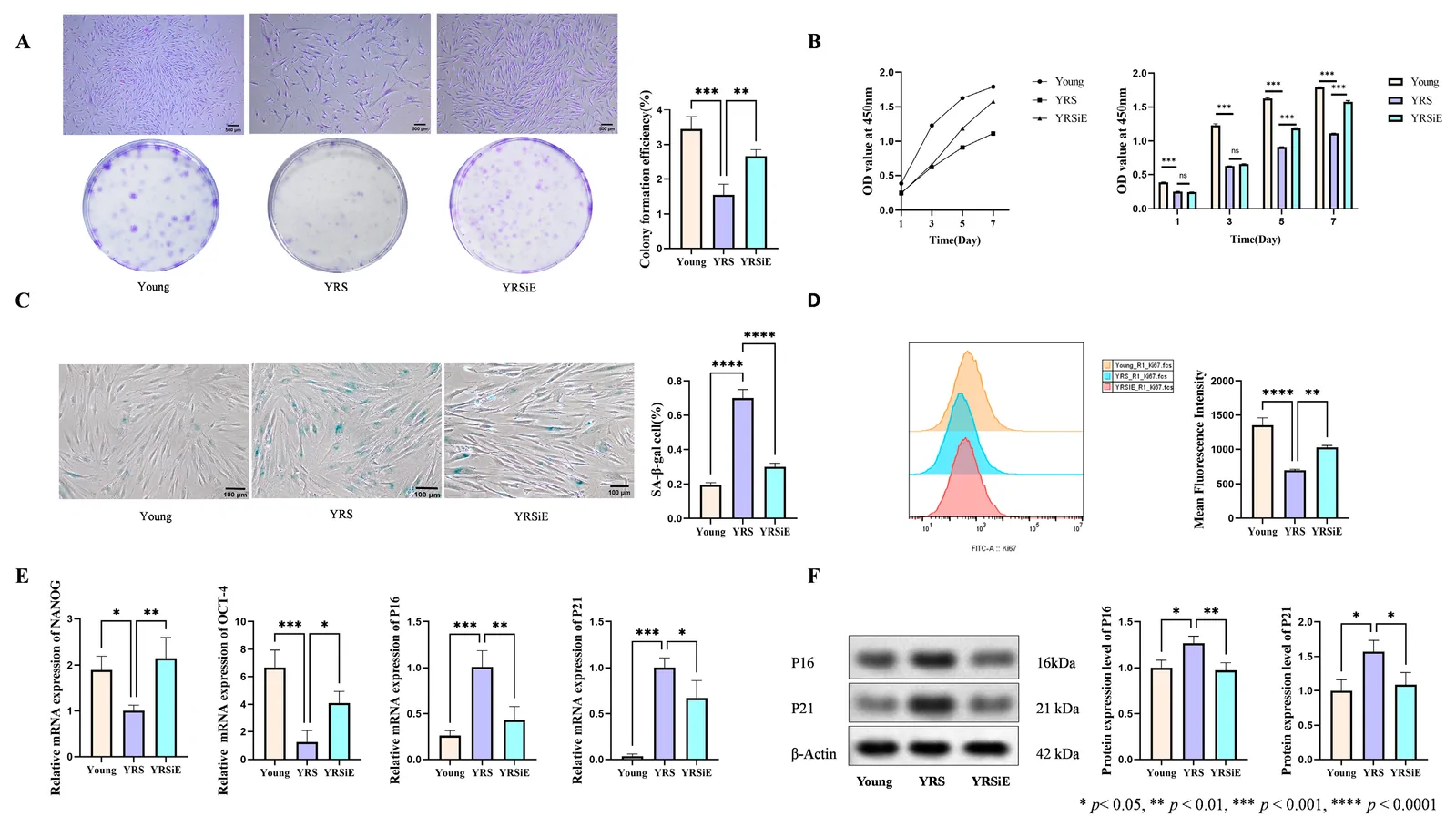
Functional effects of hiPSC extract on senescent AB-MSCs. (**A**) Colony formation assay showing a higher number of crystal violet-stained colonies in the Young group compared to the YRS and YRSiE groups (scale bar = 500 μm). Colony-forming ability was partially restored in YRSiE relative to YRS (**left**). Quantitative analysis of the colony formation efficiency (**right**). (**B**) CCK-8 assay demonstrating cell proliferation-associated metabolic activity over 7 days. The Young group exhibited the highest metabolic activity. From day 5 onward, YRSiE showed significantly increased metabolic activity compared to YRS. (**C**) SA-β-gal staining revealed the highest percentage of senescent cells in the YRS group, which was significantly reduced in the YRSiE group (scale bar = 100 μm). (**D**) Flow cytometric analysis of FITC-Ki67 staining. (**E**) qRT-PCR analysis of pluripotency associated genes (NANOG, OCT 4) and senescence related genes (P16, P21). (**F**) Western blot analysis and densitometric quantification of senescence related proteins P21 and P16. *n* = 3 donors. One-way ANOVA (**A**,**C**–**F**) and two-way ANOVA (**B**) with Tukey’s post hoc test.

Crystal violet staining revealed significantly more clonal colonies in the Young group compared to the other two groups. The YRSiE group exhibited a marked increase in colony number relative to the YRS group, in which most cells failed to form proliferative clusters (Figure 3A, left). Quantitative analysis of the colony formation assay demonstrated that the colony formation efficiency in the YRSiE group was significantly elevated compared with that in the YRS group (Figure 3A, right).

CCK-8 assay showed the highest proliferation-associated metabolic activity in the Young group over the 7 day culture. From day 5 to day 7, the proliferation curve of the YRSiE group surpassed that of the YRS group, indicating enhanced cell proliferation-associated characteristics following hiPSC extract treatment (Figure 3B).

SA-β-gal staining demonstrated the highest proportion of β-galactosidase positive cells in the YRS group, which was significantly reduced in the YRSiE group, with the Young group showing the lowest level (Figure 3C, left). Statistical analysis of the SA β gal-positive cells was shown in the right panel of Figure 3C.

Flow cytometric analysis of FITC-Ki67 staining (Figure 3D) showed that the YRS group exhibited the lowest mean fluorescence intensity (MFI) among the three groups, whereas the MFI values in both the YRSiE and Young groups were significantly higher. In conjunction with the β-galactosidase staining results, these findings indicate that YRS cells were during a senescent state, whereas hiPSC extract treatment (YRSiE) effectively attenuated this senescence.

qRT-PCR analysis indicated that expression of pluripotency associated genes NANOG and OCT 4 was lowest in the YRS group and elevated in the other two groups. In contrast, senescence related genes P21 and P16 were most highly expressed in the YRS group, while both the Young and YRSiE groups showed markedly lower levels (Figure 3E).

Western blot analysis revealed that senescence related proteins P21 and P16 were most highly expressed in the YRS group, while both the Young and YRSiE groups showed lower levels (Figure 3F).

### 3.4. hiPSC Extract Facilitates Efficient Osteogenic Differentiation of Senescent AB-MSCs In Vitro

**Figure 4:**
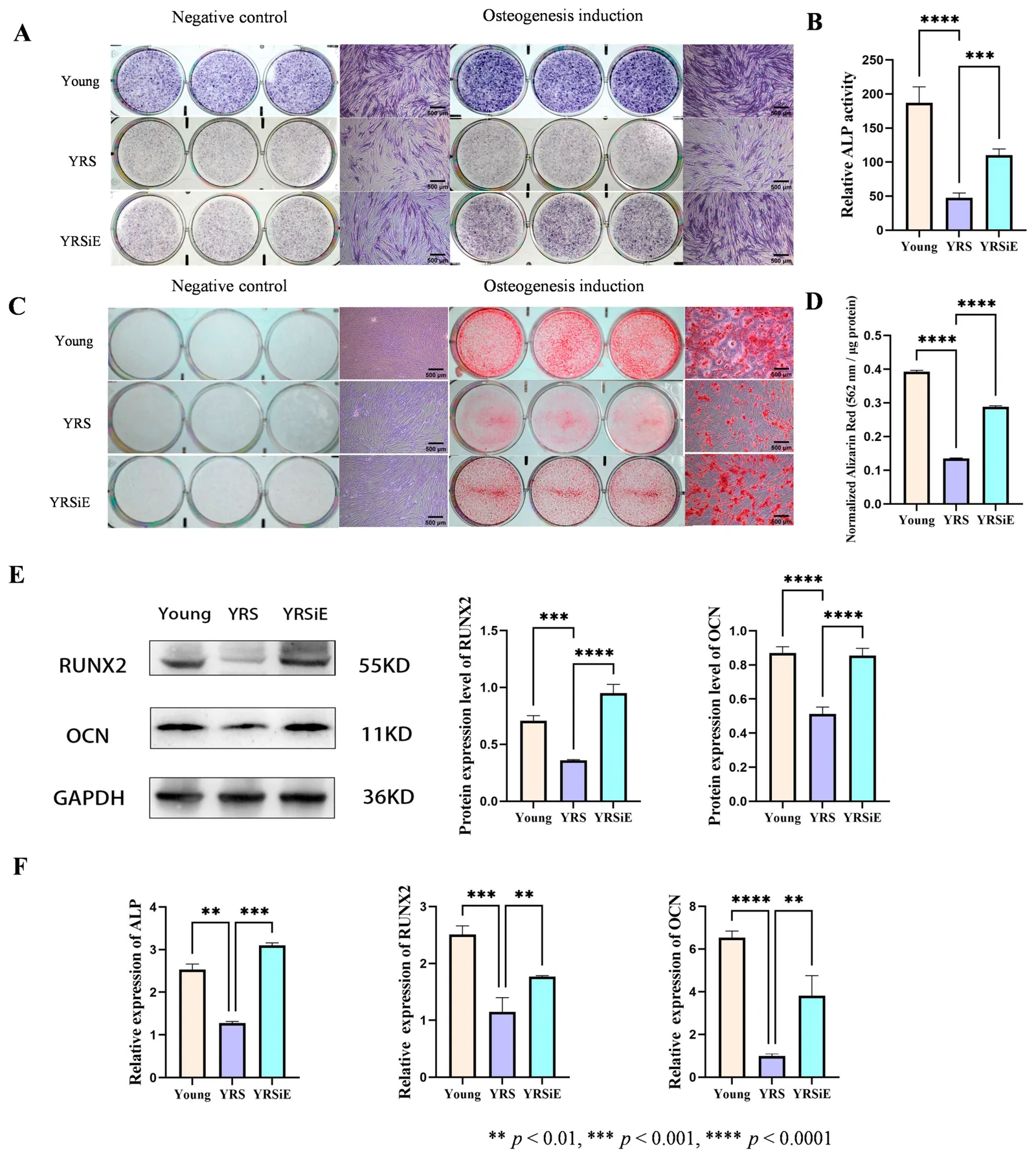
Evaluation of osteogenic differentiation capacity in AB-MSCs following treatment with hiPSC extract. (**A**) Alkaline phosphatase (ALP) staining (general and microscopic views; scale bar = 500 μm). (**B**) Quantitative analysis of ALP activity. (**C**) Alizarin Red S staining of mineralized nodules (general and microscopic views; scale bar = 500 μm). (**D**) Quantitative analysis of Alizarin Red staining. (**E**) Protein expression levels of osteogenic markers (RUNX2, OCN) analyzed by Western blot, with GAPDH as loading control. (**F**) mRNA expression levels of osteogenesis-related genes (ALP, RUNX2, OCN) measured by qRT-PCR. *n* = 3 donors. One-way ANOVA with Tukey’s post hoc test.

After seven days of osteogenic induction, alkaline phosphatase (ALP) staining and activity assays demonstrated strongest ALP expression and highest activity in the Young group. The YRSiE group showed more intense staining and significantly higher ALP activity than the YRS group (Figure 4A,B).

Following 21 days of induction, alizarin red staining detected no mineralized nodules in the blank control group. OD values were normalized to total protein content for each sample to account for differences in cell number. The Young group showed the most intense staining and the highest normalized Alizarin Red absorbance. The YRSiE group showed significantly higher normalized Alizarin Red absorbance than the YRS group (Figure 4C,D).

The protein expression levels of the osteogenic markers RUNX2 and OCN (Figure 4E) and the mRNA expression levels of ALP, RUNX2, and OCN (Figure 4F) were lowest in the YRS group. Expression of these proteins and genes was significantly elevated in the YRSiE group relative to the YRS group.

### 3.5. Differential mRNA Analysis of AB-MSCs in Young, YRS and YRSiE Groups

**Figure 5:**
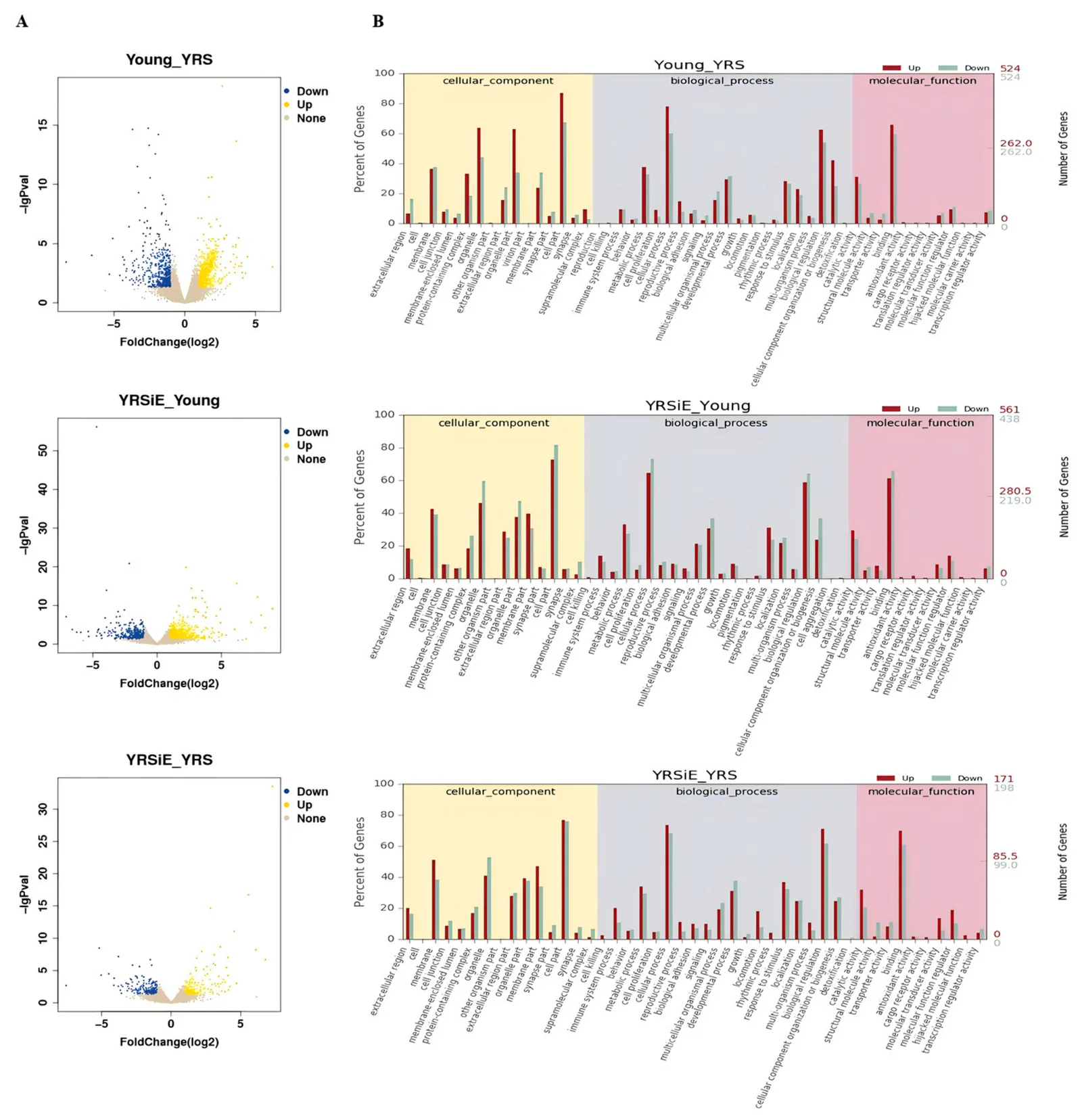
Transcriptomic profiling and functional enrichment of differentially expressed genes (DEGs) in AB-MSCs. (**A**) Volcano plots showing DEGs across the three experimental groups: Young vs. YRS, YRSiE vs. Young, and YRSiE vs. YRS. Yellow and blue dots represent up and down-regulated genes, respectively (log_2_FC ≥ 1, *q* < 0.05). (**B**) Gene Ontology (GO) enrichment analysis of common DEGs across comparisons, summarizing terms in biological process, cellular component, and molecular function categories. *n* = 3 donors. DEGs were defined as |log_2_ fold change| ≥ 1 and *q* < 0.05.

To identify target genes involved in senescence regulation, we compared mRNA expression profiles across the three AB-MSCs groups by RNA sequencing (Figure 5A). A total of 1049 differentially expressed genes (DEGs) were identified between the Young and YRS groups, including 525 up-regulated and 524 down-regulated genes in the Young group. Between the YRSiE and YRS groups, 370 DEGs were found, with 172 up-regulated and 198 down-regulated in YRSiE. Additionally, 1000 DEGs were detected between the Young and YRSiE groups, among which 562 were up-regulated and 438 down-regulated in YRSiE relative to the Young group.

Gene Ontology (GO) enrichment analysis was performed to elucidate the functional roles of these DEGs across three categories: biological process, cellular component, and molecular function (Figure 5B). The results revealed that commonly dysregulated mRNAs among the three groups were significantly associated with critical cellular functions, including “metabolic processes,” “biological regulation,” and “response to stimuli.”

**Figure 6:**
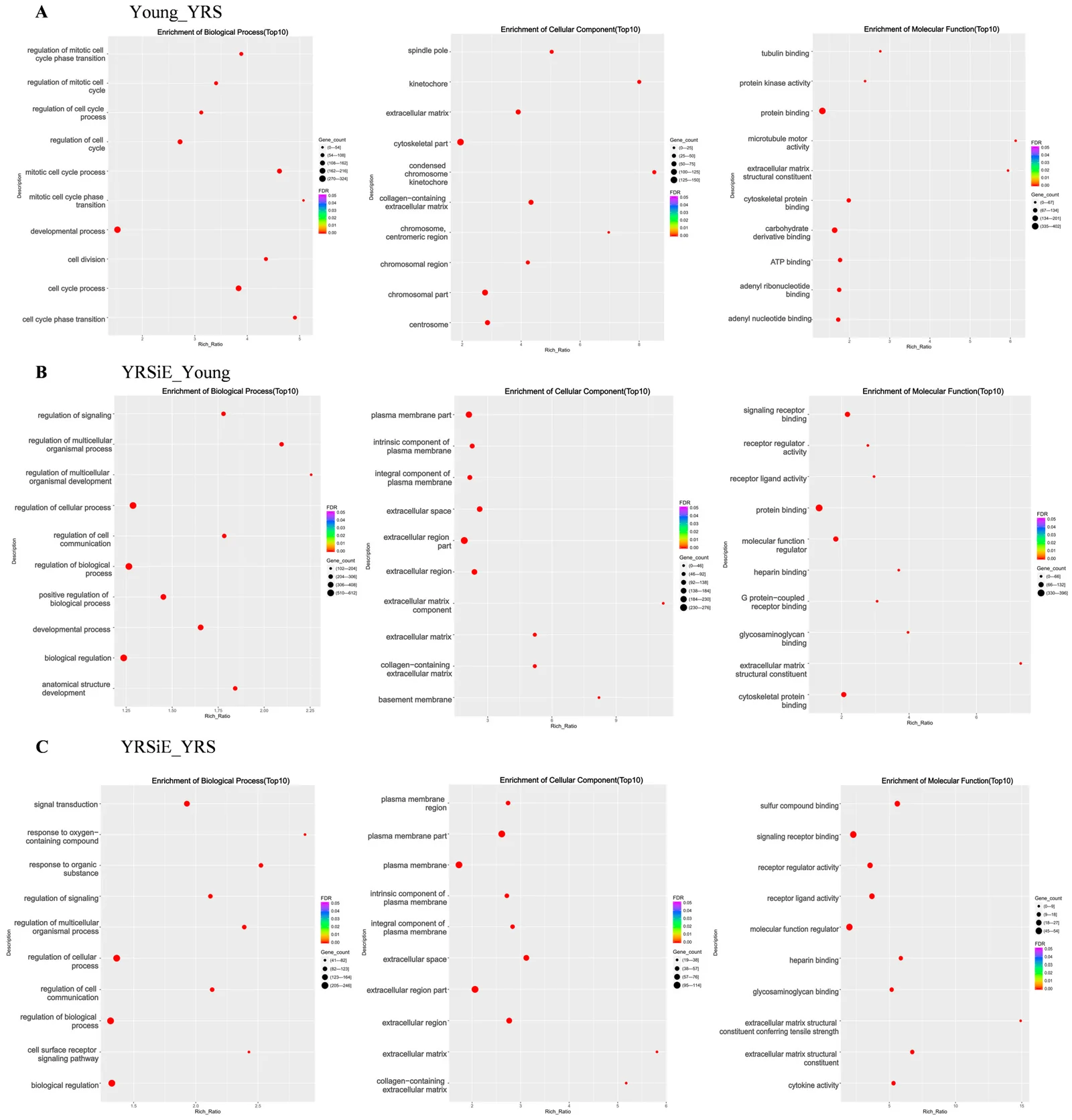
Top 10 significantly enriched Gene Ontology (GO) terms from the comparisons between (**A**) Young vs. YRS, (**B**) YRSiE vs. Young, and (**C**) YRSiE vs. YRS. Analyses were based on three biological replicates per group. *n* = 3 donors. DEGs were defined as |log_2_ fold change| ≥ 1 and *q* < 0.05.

Based on the differentially expressed genes within each Gene Ontology (GO) term, we performed enrichment analysis to evaluate their significance against the whole genome background (Figure 6). The results revealed strong associations with key biological themes including “cell cycle regulation”, “signal transduction”, and “extracellular matrix composition”. Importantly, both “cell cycle regulation” and “extracellular matrix composition” are closely related to cellular senescence.

**Figure 7:**
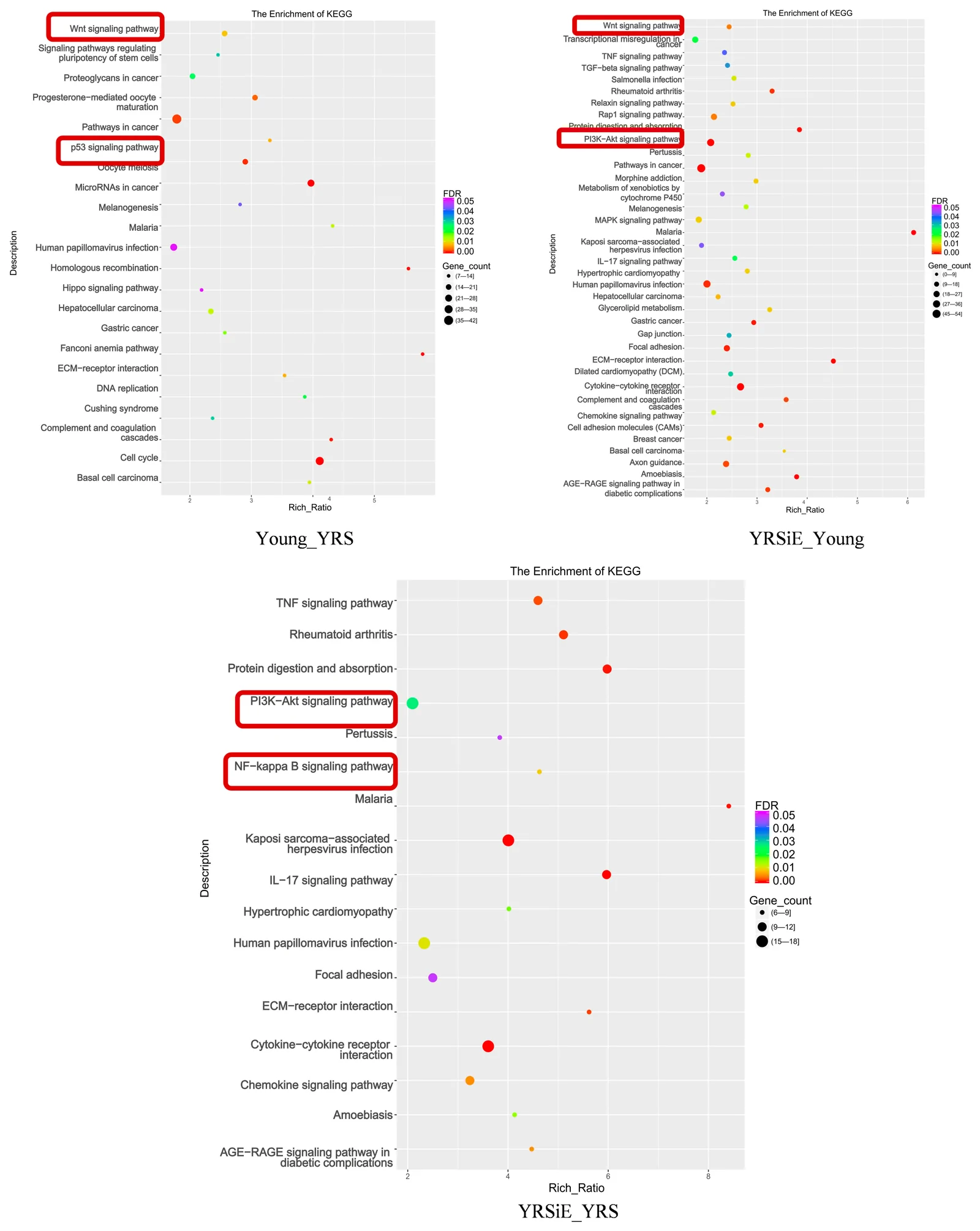
KEGG pathway enrichment analysis of differentially expressed genes across the three experimental groups. The most significantly enriched signaling pathways are labeled, with point size representing the number of genes and color indicating the statistical significance. *n* = 3 donors. DEGs were defined as |log_2_ fold change| ≥ 1 and *q* < 0.05.

KEGG enrichment analysis revealed that the differentially expressed genes among the three cell groups were involved in multiple signaling pathways (Figure 7). Among these, senescence-related pathways—including the “Wnt signaling pathway”, “NF-κB signaling pathway”, “p53 signaling pathway”, and “PI3K-Akt signaling pathway”—showed significant enrichment. These pathways are known to regulate biological processes such as stem cell pluripotency, proliferation, and cell cycle control. These results suggest that hiPSC extract treatment is associated with transcriptional changes in AB-MSCs, which may potentially contribute to the modulation of cellular senescence through these mechanisms.

### 3.6. Identification of Differentially Expressed miRNAs and NF-κB Pathway-Related Changes

**Figure 8:**
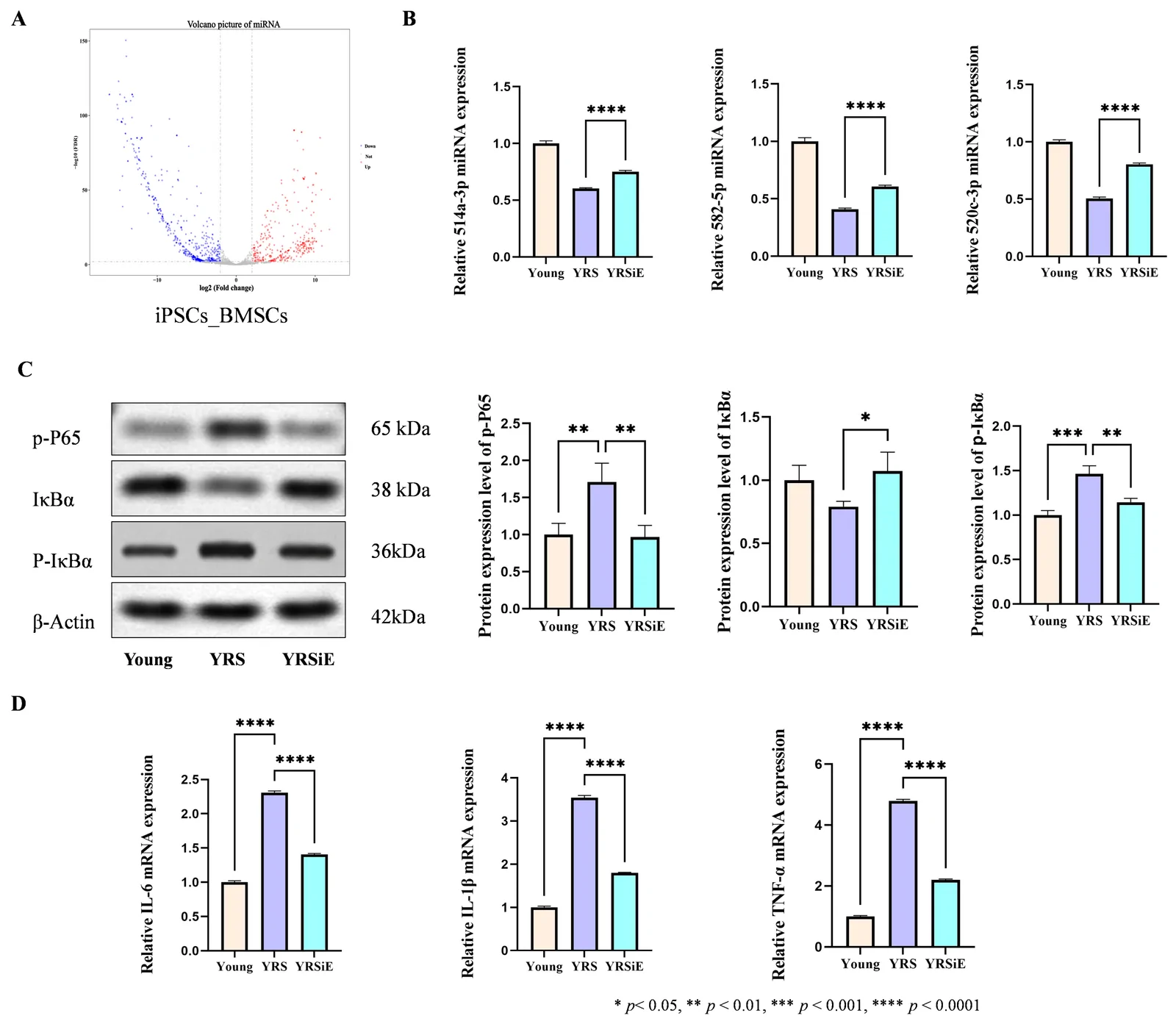
miRNA expression profiling and NF-κB pathway-related changes. (**A**) Volcano plot of differentially expressed miRNAs between AB-MSCs and hiPSCs. Red and blue dots indicate significantly upregulated and downregulated miRNAs. *n* = 3 independent donors. DEGs were defined as |log_2_ fold change| ≥ 1 and *q* < 0.05. (**B**) qRT-PCR validation of selected miRNAs. (**C**) Western blot analysis of phosphorylated p65 (p-p65), total IκBα and phosphorylated IκBα (p-IκBα) in Young, YRS and YRSiE groups. β-actin served as loading controls. (**D**) qRT-PCR analysis of NF-κB target genes IL-6, IL-1β, and TNF-α. *n* = 3 donors. One-way ANOVA with Tukey’s post hoc test.

To identify potential bioactive factors enriched in hiPSC extract, we performed small RNA sequencing on hiPSCs and AB-MSCs. A total of 881 miRNAs were differentially expressed between the two cell types, among which 338 were significantly upregulated in hiPSCs compared to AB-MSCs and 543 downregulated (Figure 8A). To further validate these findings, we performed qRT-PCR analysis in Young, YRS and YRSiE groups, which confirmed that miR-514a-3p, miR-582-5p and miR-520c-3p were significantly upregulated in the YRSiE group compared to the YRS group (Figure 8B).

Given that the mRNA sequencing analysis had identified the NF-κB signaling pathway as a significantly enriched term, the activation status of this pathway in the Young, YRS and YRSiE groups was examined. Western blot analysis revealed that, compared to the YRS group, the YRSiE group exhibited decreased phosphorylation of IκBα and p65, along with increased total IκBα protein levels. Consistently, the mRNA expression of NF-κB downstream target genes, including IL-6, IL-1β, and TNF-α, was significantly downregulated in the YRSiE group (Figure 8C,D).

## 4. Discussion

This study showed that iPSC extracts can attenuate replicative senescence in AB-MSCs, enhancing both their proliferative capacity and osteogenic potential. After prolonged in vitro culture, AB-MSCs displayed typical features of replicative senescence, such as enlarged and elongated morphology, multinucleation, and loss of three-dimensional architecture [29]. Extended treatment with iPSC extracts restored a more youthful phenotype in these senescent cells, characterized by smaller, more uniform cell size resembling that of younger AB-MSCs.

Elevated lysosomal content—a marker of senescence—was evidenced by increased β-galactosidase activity, detectable at pH < 6.0. This activity correlates with cumulative cell divisions and serves as a reliable senescence biomarker [30]. However, some studies indicate that factors other than senescence can increase SA-β-gal activity [31]. A recently proposed algorithm can more accurately distinguish between cycling, stressed, quiescent, and senescent cells [32]. Therefore, in the present study, senescent-associated cells were defined as Ki67-negative (Ki67^−^) and SA-β-gal-positive (SA-β-gal^+^). Compared with the Young and YRSiE groups, YRS cells displayed decreased Ki67 expression and increased SA-β-gal activity, indicative of cellular senescence. Notably, treatment with iPSC extracts attenuated this senescence-associated phenotype.

Senescent cells commonly exhibit irreversible cell cycle arrest, mediated by increased expression of cyclin-dependent kinase inhibitors P16 and P21 [33]. P16, a well-established senescence marker, increases with age and promotes cell cycle arrest via enhanced hypophosphorylation of retinoblastoma protein (pRB), influencing processes such as aging, cancer, and tissue regeneration [34]. Meanwhile, the stemness and pluripotency of mesenchymal stromal cells are regulated by NANOG and OCT-4, both of which decline with cellular aging [35]. NANOG helps maintain the undifferentiated state of embryonic stem cells (ESCs) and augments stemness across cell types [36]. Its overexpression enhances tumor cell proliferation and migration, while its inhibition reduces these traits [37]. OCT-4, a key transcription factor, regulates multiple microRNAs and is itself post-transcriptionally controlled; it suppresses miR-145 to preserve hESC pluripotency [38].

Following iPSC extract treatment, senescent AB-MSCs showed reduced P16 and P21 expression, indicating a reduction in senescence-associated markers, together with markedly increased NANOG and OCT-4 expression, suggesting increased expression of pluripotency-associated genes. However, these changes observed in recipient cells after extract treatment, which may reflect a combination of extract-derived RNA and cellular responses.

Reversing aging and improving bone regeneration remain critical clinical challenges [39]. Osteogenic differentiation is essential for bone formation, with mineralized nodule formation being a hallmark of maturity [40]. These nodules are commonly detected using Alizarin Red staining, which binds calcium salts to produce a characteristic red precipitate [41]. Key osteogenic genes—such as ALP, RUNX2, and OCN—show stage-specific expression patterns during this process [42]. Among them, RUNX2 is a master transcription factor stimulating mineralization and regulating genes involved in osteogenesis, chondrogenesis, angiogenesis, and odontogenesis [43]. ALP is an early osteogenic marker, while OCN indicates late osteoblast differentiation [44]. This study found that hiPSC extract increased ALP, RUNX2, and OCN expression in senescent AB-MSCs and may promote mineralized nodule formation, confirming enhanced osteogenic capacity.

KEGG and GO enrichment analyses indicated that hiPSC extract modulated several senescence-related pathways in AB-MSCs, including “PI3K-Akt signaling”, “NF-κB signaling”, “cell cycle regulation”, and “extracellular matrix composition”. Multiple pathways may contribute to the effects of hiPSC extract, and that NF-κB was selected as one representative pathway rather than as the sole mechanism. NF-κB is a key transcription factor implicated in aging-related diseases [45], modulating immune response, senescence, apoptosis, and metabolism [46]. In senescent cells, the NF-κB signaling pathway is activated, accompanied by elevated expression of senescence markers and increased secretion of senescence-associated secretory phenotype (SASP) factors. Conversely, inhibition of this pathway can effectively reverse the senescent phenotype [47]. In this study, treatment with hiPSC extract (YRSiE cells) was accompanied by lower p-p65 and p-IκBα and higher IκBα levels, changes consistent with reduced NF-κB pathway-related signaling [48]. Furthermore, qPCR analysis of well-established NF-κB downstream target genes was performed, including IL-6, IL-1β, and TNF-α. The results showed that these pro-inflammatory cytokines were higher in YRS cells, whereas their expression was lower in the YRSiE group, approaching the levels observed in Young cells. These findings are consistent with the NF-κB pathway enrichment predicted by our KEGG analysis. Several of the miRNAs upregulated in hiPSCs, including miR-514a-3p [49], miR-582-5p [50] and miR-520c-3p [51], have been previously reported to inhibit NF-κB signaling by targeting key components of this pathway, such as p50 [49], *Skp1* and p65 [51]. These hiPSC-enriched miRNAs may be candidate bioactive components of the hiPSC extract. Notably, the hiPSC extract is a complex mixture that comprises not only miRNAs but also proteins, lipids, and metabolites, all of which may contribute to the observed effects. Accordingly, the identified miRNAs should be considered candidate bioactive constituents rather than the exclusive mediators of the extract’s activity.

This study has several limitations. The causal roles of the identified miRNAs were not tested, future loss- and gain-of-function experiments are needed. The functional requirement for NF-κB activation was not directly tested, and the pathway-level association observed here should be interpreted as correlative. In vivo validation of these findings remains to be performed in future studies. The direct characterization of the hiPSC extract will be an important direction for future work. A non-pluripotent extract control and RNase-treated extract would be required in further research to formally separate endogenous transcriptional changes and direct contributions from residual extract-derived RNA. Although hiPSC extract’s composition may be relatively consistent, the variable components arising from different laboratory practices and culture conditions may lead to substantial differences in the biological activity of the extract. Therefore, the effects report here were observed under specific and well-defined culture conditions and may not be directly generalizable to other culture systems. Future studies are needed to systematically evaluate the reproducibility across diverse culture systems, which would provide a critical foundation for protocol standardization and future translational development. In addition, since oral MSCs show donor-dependent heterogeneity in CD105 expression while low-CD105 subpopulations retain MSC features [52], we characterized CD73, CD90, CD45, and CD34 to confirm plastic adherence and exclude hematopoietic contamination. HLA-DR was not examined, as this study focuses on senescence and osteogenic function rather than immunogenicity. Only osteogenic and adipogenic differentiation were assessed because AB-MSCs possess preferential osteogenic potential with weak chondrogenic capacity [53]. The complete immunophenotyping and full trilineage differentiation validation were not achieved here, and these characterizations will be supplemented in future investigations.

## 5. Conclusions

This study showed that hiPSC extract treatment attenuated senescence-associated phenotypes in AB-MSCs, accompanied by higher clonogenic ability, proliferation-associated characteristics, and osteogenic differentiation in senescent cells. Integrative miRNA and mRNA sequencing identified miR-514a-3p, miR-582-5p and miR-520c-3p as upregulated in treated cells, and the pathway-related expression changes were consistent with NF-κB pathway enrichment. Given that these miRNAs have been reported to regulate NF-κB signaling, they are candidate components of the effects of hiPSC extract. These findings provide preliminary evidence that hiPSC extract can modulate senescence-associated phenotypes in AB-MSCs in vitro, and warrant further validation of the causal roles of the candidate miRNAs and the NF-κB pathway.

## Figures and Tables

**Table 1:** Primer sequences used for qRT-PCR.

Gene	Forward Sequence (5′-3′)	Reverse Sequence (5′-3′)
GAPDH	GGAGCGAGATCCCTCCAAAAT	GGCTGTTGTCATACTTCTCATGG
NANOG	AAGGTCCCGGTCAAGAAACAG	CTTCTGCGTCACACCATTGC
OCT-4	CTTGAATCCCGAATGGAAAGGG	CCTTCCCAAATAGAACCCCCA
P16	GGGTTTTCGTGGTTCACATCC	CTAGACGCTGGCTCCTCAGTA
P21	GGCAGACCAGCCTGACAGAT	TTCAGGGTTTTCTCTTGCAGAAG
ALP	ACCACCACGAGAGTGAACCA	CGTTGTCTGAGTACCAGTCCC
RUNX2	TGGTTACTGTCATGGCGGGTA	TCTCAGATCGTTGAACCTTGCTA
OCN	CACTCCTCGCCCTATTGGC	CCCTCCTGCTTGGACACAAAG
IL-6	GTAGCCGCCCCACACAGA	CATGTCTCCTTTCTCAGGGCTG
IL-1β	TTGACGGACCCCAAAAGAT	GAAGCTGGATGCTCTCATCTG
TNF-α	ACCCTCACACTCAGATCATCTTC	TGGTGGTTTGCTACGACGT
miR-514a-3p	GATTGACACTTCTGTGAGTAG	AGTGCAGGGTCCGAGGTATT
miR-582-5p	GCACACATTGAAGAGGACAGAC	TATTGAAGGGGGTTCTGGTG
miR-520c-3p	ACACTCCAGCTGGGAAGTGCTCTTTTA	TCGCACTGGATACGACACCCTC

## Data Availability

The original contributions presented in this study are included in the article. The raw data supporting the RNA-Seq results reported in this paper have been deposited in the GSA-Human database under accession number HRA004150. Further inquiries can be directed to the corresponding authors.
